# The gut microbiome in social anxiety disorder: evidence of altered composition and function

**DOI:** 10.1038/s41398-023-02325-5

**Published:** 2023-03-20

**Authors:** Mary I. Butler, Thomaz F. S. Bastiaanssen, Caitriona Long-Smith, Sabrina Morkl, Kirsten Berding, Nathaniel L. Ritz, Conall Strain, Dhrati Patangia, Shriram Patel, Catherine Stanton, Siobhain M. O’Mahony, John F. Cryan, Gerard Clarke, Timothy G. Dinan

**Affiliations:** 1 Department of Psychiatry & Neurobehavioral Science, University College Cork, Cork, Ireland; 2 APC Microbiome Ireland, University College Cork, Cork, Ireland; 3 Department of Anatomy and Neuroscience, University College Cork, Cork, Ireland; 4 Department of Psychiatry and Psychotherapeutic Medicine, Medical University of Graz, Graz, Austria; 5 Teagasc Food Research Programme, Moorepark, Fermoy, Co, Cork, T12 YN60 Ireland; 6 School of Microbiology, University College Cork, Cork, Ireland

**Keywords:** Psychiatric disorders, Neuroscience

## Abstract

The microbiome-gut-brain axis plays a role in anxiety, the stress response and social development, and is of growing interest in neuropsychiatric conditions. The gut microbiota shows compositional alterations in a variety of psychiatric disorders including depression, generalised anxiety disorder (GAD), autism spectrum disorder (ASD) and schizophrenia but studies investigating the gut microbiome in social anxiety disorder (SAD) are very limited. Using whole-genome shotgun analysis of 49 faecal samples (31 cases and 18 sex- and age-matched controls), we analysed compositional and functional differences in the gut microbiome of patients with SAD in comparison to healthy controls. Overall microbiota composition, as measured by beta-diversity, was found to be different between the SAD and control groups and several taxonomic differences were seen at a genus- and species-level. The relative abundance of the genera *Anaeromassillibacillus* and *Gordonibacter* were elevated in SAD, while *Parasuterella* was enriched in healthy controls. At a species-level, A*naeromassilibacillus sp An250* was found to be more abundant in SAD patients while *Parasutterella excrementihominis* was higher in controls. No differences were seen in alpha diversity. In relation to functional differences, the gut metabolic module ‘aspartate degradation I’ was elevated in SAD patients. In conclusion, the gut microbiome of patients with SAD differs in composition and function to that of healthy controls. Larger, longitudinal studies are warranted to validate these preliminary results and explore the clinical implications of these microbiome changes.

## Introduction

Social anxiety disorder (SAD) is one of the most common psychiatric conditions with estimated lifetime prevalence rates as high as 13% reported in the United States [1] and similarly high prevalence rates across Europe [2]. The current Diagnostic and Statistical Manual of Mental Disorders – Fifth Edition (DSM-V) describes SAD as a condition characterised by ‘marked fear or anxiety about one or more social situations in which the individual is exposed to possible scrutiny by others.’ These fears generally extend across a variety of situations, including social interactions (e.g., having a conversation, meeting unfamiliar people), being observed (e.g., eating or drinking), and performing in front of others (e.g., giving a speech) [3]. SAD typically begins early in life and tends to run a chronic, often lifelong, course [4]. It is associated with serious functional disability and markedly reduced quality of life [5] with up to 69% of sufferers experiencing another lifetime major comorbid disorder [6]. In particular, SAD markedly increases the risk of subsequent depression [7] which is associated with a poorer prognosis and greater risk of suicide attempts [8]. Thus, timely intervention in this early-onset disorder has the potential to not only reduce substantial disability, but to markedly reduce the psychiatric disease burden later in life. Current first-line treatments include selective serotonin reuptake inhibitors (SSRIs), serotonin and norepinephrine reuptake inhibitors (SNRIs) and cognitive behavioural therapy [9]. Unfortunately, a significant proportion of patients fail to adequately respond to first-line pharmacotherapy [10] and even fewer patients will respond to subsequent second-line treatments. In a large randomised controlled trial (RCT) investigating augmentation and switch strategies for refractory SAD (defined as more than two unsuccessful adequate pharmacological treatment trials), only 46% of patients demonstrated a response to treatment, while only 21% of all patients achieved remission at the 12-week endpoint [11]. Thus, there is a great necessity for an improved understanding of the neurobiological basis for this condition and the development of alternative therapeutic strategies. The microbiome-gut-brain axis may represent one such potential avenue for investigation.

The human gastrointestinal tract (GIT) harbours a vast assembly of microorganisms, predominantly bacteria but also fungi, viruses, protozoa and archaea. While the term gut microbiota refers to the assemblage of living organisms present within the gut, the term microbiome encompasses the micro-organisms and their ‘theatre of activity’ i.e., their structural elements, genomes and metabolites, as well as the surrounding environmental conditions [12, 13]. It is estimated that the number of bacteria in the human gut is slightly in excess of the total number of human cells, at approximately 3.8 ×10 ^13^ [14] and that the collective genome of these bacterial cells vastly exceeds the amount of human DNA present in the body [15]. Given this enormous, modifiable reservoir of genetic potential, it is unsurprising that there is keen interest in the potential role of the gut microbiome in the aetiology and treatment of many disease processes. The microbiome is recognised as a key player in bidirectional signalling between the gut and brain, with the term ‘microbiome-gut-brain’ (MGB) axis describing this communication network. Many physiological systems relevant to psychiatric disorders come under the influence of the gut microbiome, including the immune system, vagal neurotransmission, tryptophan metabolism, endocrine function and the stress response system, making the MGB axis an attractive new therapeutic target in psychiatry [16].

Despite a growing interest in the role of the gut microbiome in the neurobiology of the stress response [17] and social behaviour [18, 19], there has been very limited investigation of the gut microbiome in relation to SAD. Indeed, apart from a few small studies in generalised anxiety disorder (GAD) [20–22], and in co-morbid irritable bowel syndrome (IBS) [23], gut microbiome composition or function has remained largely unexplored in patients with clinical anxiety disorders, including SAD, panic disorder and agoraphobia. This is despite an abundance of preclinical studies demonstrating that anxiety-like behaviours are altered in animal models following a variety of microbiota manipulations [24] and clear evidence that certain probiotics can reduce self-reported anxiety levels in healthy human volunteers [25] The past decade has also seen a spate of preclinical studies revealing a role for the gut microbiome in social development and social behaviour in animals [26, 27] and this has extended to human studies in recent years. Differences in gut microbiota composition and diversity have recently been linked to personality traits such as sociability in the general population [28]. Additionally, altered gut microbiota composition has been demonstrated in adults experiencing social exclusion [29], a psychological phenomenon to which those with SAD are particularly sensitive [30]. Interestingly, a potential therapeutic role for the microbiota in SAD is supported by a cross-sectional study which reported that higher intake of fermented, probiotic-containing foods by healthy students appeared to be protective against developing SAD in those who were at higher genetic risk, as measured by trait neuroticism [31].

Here we report on compositional and functional differences in the gut microbiome of patients with SAD using whole-genome shotgun analysis of 49 faecal samples (31 cases and 18 controls). The functional differences, based on Kyoto Encyclopedia of Genes and Genomes (KEGG) orthologues, are explored using the recently described gut-brain module (GBM) [32] and gut metabolic module (GMM) [33] analysis. We hypothesise that the gut microbiome is compositionally and functionally altered in those with SAD.

## Methods

### Participants

Patients with a clinical diagnosis of SAD were recruited through local general practitioners, psychologists and outpatient psychiatric clinics. The study was also advertised through local and national SAD support groups and by an online website (www.sadgut.ie). Eligibility was limited to men and women aged between 18–65 years with a clinical diagnosis of SAD. Exclusion criteria included any significant acute or chronic medical illness (including functional gastrointestinal disorders such as irritable bowel syndrome); the presence of any condition or medication which the investigator believed would interfere with the objectives of the study or confound the interpretation of the study, including anticonvulsants, centrally acting corticosteroids, opioid pain relievers, laxatives, enemas, anti-coagulants, over-the counter non-steroidal anti-inflammatories (NSAIDS); the use of probiotics, prebiotics or antibiotics in the previous 4 weeks; females who were pregnant or breastfeeding; subjects who were vegetarian or adhering to a strict specific diet. Specific psychiatric exclusion criteria included a lifetime diagnosis of psychotic disorder, intellectual disability, bipolar disorder, dementia or ASD and a current diagnosis of major depressive disorder (MDD), eating disorder, alcohol or substance abuse or dependence. A past history of depression was permitted, as was the presence of a comorbid anxiety disorder, provided the clinician was satisfied that SAD was the primary diagnosis. SAD participants were permitted to continue taking their regular psychotropic medication. Healthy controls were recruited through email and print advertising in University College Cork. Controls were required to have no past or current psychiatric diagnosis along with the other general exclusion criteria outlined for the SAD participants.

### Procedures

All study procedures were approved by the Clinical Research Ethics Committee of the Cork Teaching Hospitals (Study number APC085) and the study was conducted in accordance with the ICH Guidelines on Good Clinical Practice, and the Declaration of Helsinki. All participants provided written informed consent.

All participants were interviewed by an experienced psychiatrist using the MINI International Neuropsychiatric Interview (Version 7.0) [34] to confirm the diagnosis of SAD based on DSM-V criteria and assess for any relevant comorbidities. No patient met the criteria for the ‘performance only’ specifier and all experienced social anxiety symptoms across a range of situations.

Social anxiety symptoms were assessed using the Liebowitz Social Anxiety Scale-Self Report (LSAS), a 24-item scale which was initially developed as a clinician-rated instrument [35, 36] but was later shown to have excellent psychometric properties as a self-report scale [37–39]

To quantify nutrient intake, participants completed the self-administered 152-item SLAN-06 (Survey of Lifestyle, Attitudes and Nutrition in Ireland) food frequency questionnaire (FFQ), which is adapted from the EPIC Norfolk questionnaire [40] and validated to be used in an Irish population [41]. Participants were asked to estimate the frequency with which they consumed a specified portion size of each of the foods listed over the preceding year. The FFQs were analysed for nutrient intake using the FETA software [42]. Stool consistency was assessed using the Bristol Stool Chart (BSC) [43]. Exercise levels were measured using the International Physical Activity Questionnaire (self-administered short form) [44] and sleep using the Pittsburgh Sleep Quality Index [45].

### Biological/faecal samples

Freshly voided faecal samples were collected from study participants into plastic containers containing an AnaeroGen sachet (Oxoid AGS AnaeroGen Compact, Fischer Scientific, Dublin) to generate anaerobic conditions within the container. Participants were instructed to collect the faecal sample as close to the study visit as possible and to keep the sample containers in a refrigerator at 4 °C until delivery to the study site. A cool pack was used to transport the sample to the study site, where it was immediately stored at − 80 °C for later analysis.

### Microbiome sample preparation and whole genome shotgun sequencing

Total bacterial metagenomics DNA was extracted using the QIAmp Fast DNA Stool Mini kit (Qiagen, UK) with a modified protocol combined with repeated bead beating method (Zhongtang Yu & Mark Morrison 2018). Briefly, 1 ml of lysis buffer (500 mM NaCl, 50 mM Tris-HCl pH8.0, 50 mM EDTA and 4% sodium dodecyl sulphate) was added to the stool sample in the bead-beating tube. The samples were homogenized using a mini beadbeater (BioSpec) and incubated at 70 °C for 15 minutes (for cell lysis) followed by centrifugation at 4 °C. The supernatant was removed and the bead-beating step was repeated. Ammonium acetate (Sigma Aldrich, Ireland) was added to the pooled supernatant and incubated on ice. Following a centrifugation step the supernatant was transferred to Eppendorf tubes containing iso-propanol. The following day, DNA was pelleted and washed with 70% ethanol and dissolved in Tris-EDTA. The DNA was then RNAse and proteinase-K treated and purified according to the manufacturer’s instructions (QIAmp Fast DNA Stool Mini kit; Qiagen, UK). The DNA was quantified using Qubit and stored at −30 °C.

Whole genome shotgun sequencing was performed using Nextera XT kit. Library prep was done following the Nextera XT DNA Library Preparation Guide from Illumina. Quality of the library was evaluated using the Agilent High Sensitivity DNA chip and running it on the Bioanalyzer and the DNA was quantified using Qubit DNA High sensitivity kit read on a qubit fluorometer 3.0. The samples were pooled and sequencing was carried out on the NextSeq500 using a 300 cycle High Output v2 kit.

### Taxanomic and functional analysis

We performed quality checks on raw sequences from all faecal samples using FastQC [46]. Shotgun metagenomic sequencing data were then processed through analysis workflow that utilizes Huttenhower Biobakery pipeline [47], including Kneaddata [48], MetaPhlAn3 [49] and HUMAnN3 [50] to obtain species, genes and pathways abundance matrix. Briefly, quality filtering and host genome decontamination (human) was performed using Trimmomatic [51] and Bowtie2 [52] via Kneaddata wrapper program with following parameters: ILLUMINACLIP:/NexteraPE-PE.fa:2:30:10, SLIDINGWINDOW:5:25, MINLEN:60, LEADING:3, TRAILING:3. Taxonomic and functional profiling of the microbial community was performed using MetaPhlan3 and HUMAnN3 using default parameter. Next, gene abundance matrix was further collapsed by KEGG Orthology (KO) term and Gene Ontology (GO) term mapping via “humann_regroup_table” function provided within HUMAnN3.

Further data-handling was undertaken in R (version 4.03) using the Rstudio GUI (version 1.4.1103). In all microbiome analysis with the exception of alpha diversity, taxa with a prevalence of <5% of samples at the genus level were excluded from analysis as ratios are invariant to sub-setting and this study employs compositional data analysis techniques [53, 54]. Principal component analysis was performed on centred log-ratio transformed (clr) values using the ALDEx2 library [55]. The number of permutations was always set to 1000. Beta diversity was computed in terms of Aitchison distance, or Euclidean distance between clr-transformed data. Alpha diversity was computed using the iNEXT library [56]. KEGG orthologues were used as features to compute functional alpha diversity. Gut-Brain Modules (GBMs) and Gut-Metabolic Modules (GMMs) were calculated from HUMAnN3 output using the R version of the Gomixer tool [32]. Stacked barplots were generated by normalising counts to 1, generating proportions. Genera that were never detected at a 10% relative abundance or higher were aggregated and defined as rare taxa for the purposes of the stacked barplots. Differential abundance of both microbes and functional modules were calculated using implementations of the ALDEx2 library. Effect sizes were calculated using Cohen’s D statistic. A *p* value of <0.05 was deemed significant in all cases. To correct for multiple testing in tests involving microbiome features, the Benjamini-Hochberg (BH) post-hoc was performed with a q-value of 0.1 used as a cut-off for species and 0.2 for functional modules. Plotting was done using the *ggplot2* [57] and *patchwork* [58] libraries in R. Custom R scripts and functions are available online at https://github.com/thomazbastiaanssen/Tjazi [59]. A linear modelling approach was used to test for a group effect on taxonomic and functional differences, whilst adjusting for covariates including age, sex, BMI, exercise and dietary differences.

### Statistical analysis of metadata

All metadata were analysed using SPSS 25 (IBM, Armonk, NY, USA). Visual inspection of box plots was used to identify outliers and consideration given to removal of those lying more than three times the interquartile range (IQR) below the first quartile or above the third quartile. Missing values were excluded from analysis. Normality of data was assessed by visual inspection of histograms along with examination of skewness and the Shapiro-Wilk statistic. Differences in demographic data, FFQ and LSAS scale scores between the SAD and control group were assessed using Chi-squared or Fisher’s Exact test for categorical variables, and independent t-tests or non-parametric Mann-Whitney U tests for continuous variables. Data are presented as mean ± SD unless stated otherwise.

## Results

### Demographics

Based on previous microbiota studies from our laboratory [60], a sample size of 30 participants was estimated to achieve significant changes in microbiota composition. Thirty-one patients with social anxiety disorder (SAD) and eighteen healthy controls participated in the study. There were no significant differences between patients and controls in relation to age, sex, race, years of education, birth delivery mode, alcohol consumption, smoking status, or stool consistency (Table 1). Individuals in the SAD group had higher BMI scores compared to controls (t(46)=2.65, *p* = 0.01). SAD patients had significantly lower exercise levels than controls, based on mean IPAQ scores (t(45) = −2.125, *p* = 0.04), the difference when looking at exercise categories being at the level of a trend (X^2^(2) = 5.822, *p* = 0.054). Almost three-quarters (74.2%) of SAD patients had a past history of MDD and 35.5% had a comorbid secondary anxiety disorder. Just over two-thirds (67.7%) of patients were taking psychotropic medication. The majority of these patients were prescribed an SSRI (48.4%) (8 taking Escitalopram, 2 taking Vortioxetine, 2 taking Sertraline, 1 taking Citalopram, 1 taking Paroxetine, 1 taking Fluoxetine) or SNRI (9.7%) (3 taking Venlafaxine) with 22.6% (*n* = 7) taking an alternative regular psychotropic medication (1 patient taking Pregabalin, 1 taking Agomelatine, 1 taking Bupropion, 2 taking Trazadone and 2 taking low-dose (50 mg) Quetiapine).

**Table 1:** Demographic Characteristics and Psychological Scales.

	SAD (*n* = 31)	Controls (*n* = 18)	*p* value
Age (years); mean (SD)	36.0 (11.96)	41.7 (10.79)	0.10
Gender; % female (*n*)	48.4 (15)	66.7 (12)	0.25
Race; % Caucasian (n)	100 (31)	94.4 (17)	0.37
Years of Education; mean (SD)	17.32 (4.32)	19.25 (4.89)	0.16
Delivery mode at birth			
• Vaginal	74.2 (23)	83.3 (15)	0.74
• Caesarean section	6.5 (2)	5.6 (1)	
• Unknown	19.3 (6)	11.1 (2)	
BMI (kg/m^2^); mean (SD)	28.02 (5.0)	24.22 (4.49)	0.01 *
Alcohol (units per week); mean (SD)	5.52 (7.4)	2.63 (3.1)	0.78
Alcohol categories; % (*n*)			
• 0–3 units/week	58.1 (18)	72.2 (13)	0.21
• 4–9 units/week	16.1 (5)	22.2 (4)	
• ≥ 10 units/week	25.8 (8)	5.6 (1)	
Smoking status; % smokers (*n*)	12.9 (4)	5.6 (1)	0.64
Exercise (IPAQ score); mean (SD)	3143.89 (3769.34)	5816.17 (4650.98)	0.04 *
Exercise (IPAQ category); % (n)			
• Low	25.8 (8)	5.6 (1)	0.054
• Moderate	35.5 (11)	16.7 (3)	
• High	38.7 (12)	66.7 (12)	
Bristol Stool Chart; % (n)			
• Score 1	0 (0)	0 (0)	0.50
• Score 2	22.6 (7)	11.1 (2)	
• Score 3	32.3 (10)	38.9 (7)	
• Score 4	29.0 (9)	38.9 (7)	
• Score 5	6.4 (2)	11.1 (2)	
• Score 6	9.7 (3)	0 (0)	
**Comorbidity: % (*n*)**			
Past history of MDD	74.2 (23)	0 (0)	<0.0005 *
Other anxiety disorder (total)	35.5 (11)	0 (0)	
• Agoraphobia	22.6 (7)		<0.0005 *
• GAD	6.5 (2)		
• Panic Disorder	3.2 (1)		
• Multiple	3.2 (1)		
**Psychotropic medication % (*n*)**			
• No medication	32.3 (10)	100 (18)	<0.0005 *
• Taking medication	67.7 (21)	0 (0)	
◦ SSRI	48.4 (15)		
◦ SNRI	9.7 (3)		
◦ Other regular anxiolytic	22.6 (7)		
◦ As required Beta-blocker	12.9 (4)		
◦ As required Benzodiazepine	6.4 (2)		
**Social anxiety scale scores**			
LSAS; mean (SD)			
• Fear Subscale	44.94 (11.91)	10.67 (10.31)	<0.0005 *
• Avoidance subscale	38.16 (13.47)	8.33 (10.54)	<0.0005 *
• Social interaction subscale	39.13 (13.19)	9.2 (10.38)	<0.0005 *
• Performance subscale	43.68 (14.04)	9.67 (9.71)	<0.0005 *
• Total	83.13 (24.77)	19 (19.61)	<0.0005 *

*BMI* Body Mass Index, *GAD* Generalised Anxiety Disorder, *IPAQ* International Physical Activity Questionnaire, *LSAS* Liebowitz Social Anxiety Scale, *MDD*: Major Depressive Disorder, *PSQI* Pittsburgh Sleep Quality Index, *SAD* Social Anxiety Disorder, *SD* Standard Deviation, *SNRI* Serotonin and Norepinephrine Reuptake Inhibitor, *SSRI* Selective Serotonin Reuptake Inhibitor

*p* values based on Fisher’s exact test for categorical variables and Student’s T-tests for all continuous variables apart from alcohol (units per week) which was a nonparametric Mann-Whitney U test. p-values reaching statistical significance are accompanied by an asterisk.

#### Dietary intake

Based on FFQ analysis, the only significant difference in nutrient intake seen between the SAD patients and controls was in relation to carbohydrates (Table 2). SAD patients had greater intake of total carbohydrates (U = 154, z = −1.983, *p* = 0.047), which appeared to be driven by higher total sugar intake (U = 140, z = −2.31, *p* = 0.021) as other carbohydrate groups, starch and fibre, were equivalent in patients and controls. No other differences in nutrient groups, vitamins or minerals were seen between patients and controls.

**Table 2:** Dietary intake (median (IQR)) obtained from analysis of food frequency questionnaires.

	Recommended daily intake*	Control	SAD	*p* value
		Median (IQR)	Median (IQR)	
		(% total energy)	(% total energy)	
Kilocalories	2000–2400 (males; depending on activity level)	1493 (1011)	2255 (1771)	0.12
Protein (g)	10–35% of total energy	69 (41) (18.5%)	90 (58) (16%)	0.17
Fat (g)	20–35% of total calories	60 (35) (36%)	67 (78) (27%)	0.66
Carbohydrate (g)	45–65% of total calories	171 (123) (46%)	258 (276) (46%)	0.047 *
Monounsaturated fatty acids (g)	>12% of total energy	25 (14) (15%)	26 (28) (10%)	0.45
Polyunsaturated fatty acids (g)	>6% of total energy	12 (11) (7%)	13 (15) (5%)	0.91
Saturated fatty acids (g)	<10% of total energy	20 (15) (12%)	21 (27) (8%)	0.52
Cholesterol (mg)	300 mg	225 (170)	251 (153)	0.58
Total sugar (g)	<10% of total energy	80 (70) (21%)	118 (87) (21%)	0.02 *
Starch (g)		107 (79)	159 (171)	0.08
Fibre (g)	>25 g	16 (20)	21 (21)	0.60
Vitamin A (µg)	800 µg	291 (686)	189 (245)	0.48
Thiamine (mg)	1.1 mg	1.6 (1.1)	2 (1.8)	0.12
Riboflavin (mg)	1.4 mg	1.3 (1)	1.6 (2.8)	0.17
Niacin (mg)	16 mg	22.9 (15.7)	31.5 (25.8)	0.10
Vitamin B6 (mg)	1.4 mg	2.3 (1.3)	2.9 (3)	0.17
Vitamin B12 (µg)	2.5 µg	5.5 (4.7)	5.2 (5.3)	0.60
Folate (µg)	200 µg	277 (266)	350 (445)	0.60
Vitamin C (mg)	80 mg	90 (125)	120 (121)	0.61
Vitamin D (µg)	5 µg	2.7 (3.4)	2.8 (4.2)	0.41
Vitamin E (mg)	12 mg	9.8 (9.7)	12.8 (13.3)	0.60
Phosphorous (mg)	700 mg	1020 (681)	1310 (1402)	0.38
Calcium (mg)	1000 mg	464 (319)	547 (438)	0.78
Iron (mg)	7 mg	12 (8)	14 (20)	0.48
Selenium (µg)	55 µg	53 (38)	66 (55)	0.76
Zinc (mg)	10 mg	7.8 (4.5)	10.4 (8)	0.32
Sodium (mg)	1600 mg	2599 (1597)	2877 (2293)	0.33
Potassium (mg)	2000 mg	3043 (1742)	3422 (3731)	0.61
Magnesium (mg)	375 mg	302 (190)	321 (296)	0.50
Copper (mg)	1 mg	1.1 (0.9)	1.3 (1.4)	0.52
Chloride (mg)	800 mg	3713 (2413)	4338 (3609)	0.29
Manganese (mg)	2 mg	3.6 (2.5)	3.2 (3.1)	0.68
Iodine (µg)	15 µg	101 (59)	117 (79)	0.70

(*P*-values based on results of non-parametric Mann-Whitney U tests. *p* values reaching statistical significance are accompanied by an asterisk).

#### Compositional differences in the gut microbiota of SAD patients

**Fig. 1:**
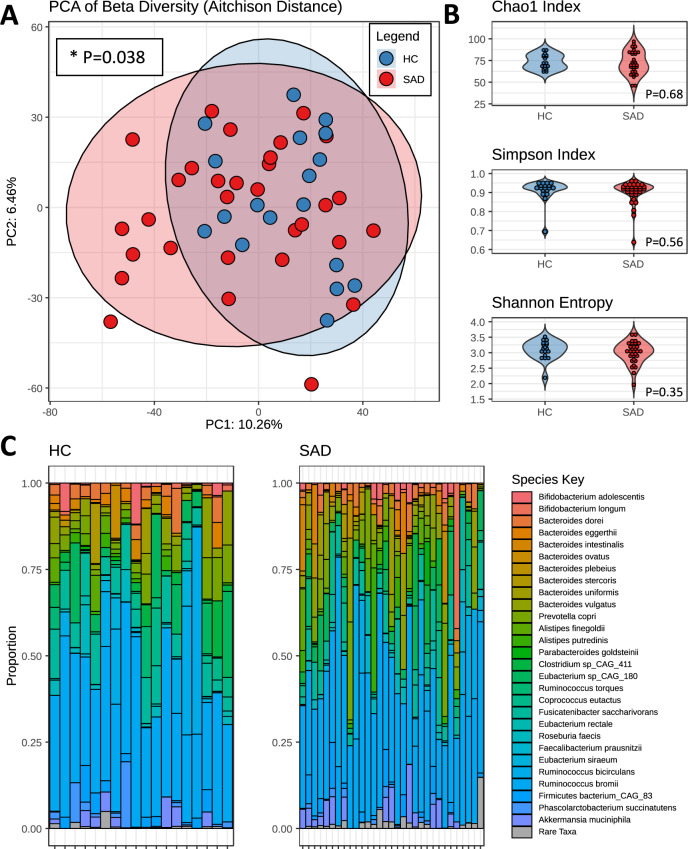
Gut Microbiota differences between SAD and control groups. **A** Beta diversity between SAD and healthy control groups, as measured by Aitchison Distance. p-value based on PERMANOVA test. **B** Alpha-diversity between SAD and healthy controls, as measured by Chao1, Simpson and Shannon indices. p-values based on Student’s t-tests. **C** Relative abundance of species-level taxa for each participant. Each column represents one participant. Genera that were never detected at a 10% relative abundance or higher are aggregated and defined as rare taxa for the purposes of the stacked barplots. (* *p* = <0.05) (HC: Healthy Control, SAD: Social Anxiety Disorder).

The gut microbiota of patients with SAD differed from those of healthy controls in terms of overall composition as well as in relation to specific genus- and species-level differentially abundant features. Beta diversity was found to be different between the two groups as measured by PERMANOVA (p = 0.038, R^2^ = 0.028) using the compositionally appropriate Aitchison distance metric (Fig. 1A). No differences were found between groups in alpha diversity, based on the Chao1, Shannon or Simpson indices (Fig. 1B).

**Fig. 2:**
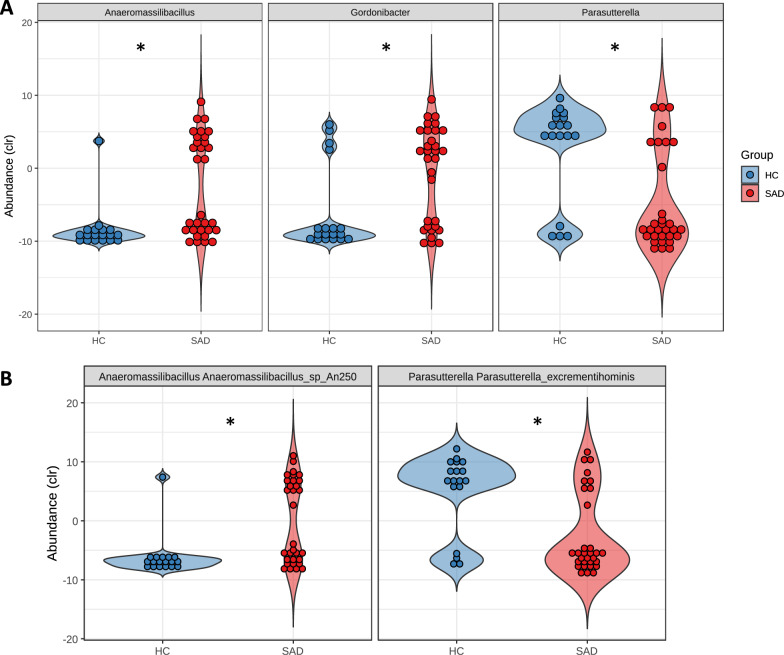
Genus and species level differences between SAD and healthy controls. **A** Genus-level differences in relative abundance between SAD and controls seen in three genera; *Anaeromassillibacillus* and *Gordonibacter* are enriched in SAD while *Parasutterella* is enriched in healthy controls. **B** Species-level differences in relative abundance between SAD and controls; *Anaeromassilibacillus sp An250* is increased in SAD while *Parasuterella excrementihominis* is enriched in healthy controls. (*p = <0.05) (Clr centred log-ratio transformed, HC Healthy Control, SAD Social Anxiety Disorder).

A total of 73 genera and 159 species were identified (Fig. 1C). Of these, three genera and two species were found to show significant differences in relative abundance after false discovery rate (FDR) correction using the Benjamini-Hochberg procedure. At the genus level, A*naeromassilibacillus* and *Gordonibacter* were enriched in SAD while *Parasutterella* was more abundant in the control samples (Fig. 2A). At the species level, A*naeromassilibacillus sp An250* was found to be more abundant in SAD patients (padj = 0.024, effect size = −1.036). Specifically, this species was present in 48.4% (15/31) of the SAD samples but only found in 5.6% (1/18) of the control samples. Conversely, the bacterial species, *Parasutterella excrementihominis* was found to be enriched in healthy controls (padj = 0.042, effect size = 1.120) (Fig. 2B). After adjusting for age, sex, BMI, exercise and dietary differences (total carbohydrates), these genus- and species-level relative abundance differences remained significant. We found no statistically significant differences in the relative abundance of any microbial taxa between unmedicated SAD patients and those taking psychotropic medication or between those SAD participants with or without a history of MDD.

#### Functional differences in the gut microbiome of SAD patients

**Fig. 3:**
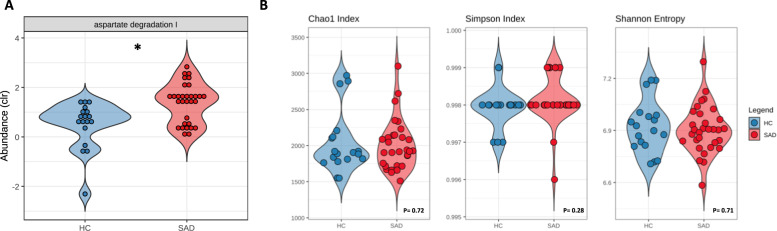
Functional differences between SAD and control groups. **A** One gut metabolic module, Aspartate Degradation I, was found to be increased in SAD patients. **B** Functional diversity, between SAD and healthy controls, as measured by Chao1, Simpson and Shannon indices. *p* values based on Student’s t-test. No differences seen between the groups. (*p = <0.05) (Clr centred log-ratio transformed, HC Healthy Control, SAD Social Anxiety Disorder).

No differences were found in functional diversity between the two groups (Fig. 3A). We identified 69 of the 103 Gut-Metabolic Modules (GMMs) curated in the current database [33] and 26 of the 56 Gut-Brain Modules (GBMs) characterised by Valles-Colomer and colleagues [32]. No GBMs reached our threshold for significance after FDR correction. However, one GMM, Aspartate Degradation I, describing the capacity of the microbiome to degrade aspartate by the enzyme aspartate aminotransferase (AspAT), was found to be significantly more abundant in patients with SAD (padj = 0.150, effect size = −1.032) (Fig. 3B). This functional difference remained between the two groups after adjusting for age, sex, BMI, exercise and dietary differences (total carbohydrates).

We then set out to investigate whether any microbial taxa or functional modules were associated with LSAS scores. After controlling the FDR, we did not detect any such associations (data not shown).

## Discussion

This study demonstrates, for the first time, that the gut microbiome is compositionally and functionally altered in people with social anxiety disorder (SAD) compared with healthy controls. Moreover, it increases the growing evidence linking social brain function and the microbiome [27]. Firstly, we show that beta diversity, an indicator of overall microbiota composition, was significantly different between the two groups. The relative abundance of three genera, A*naeromassilibacillus, Gordonibacter* and *Parasutterella*, and two corresponding species, A*naeromassilibacillus sp An250* and *Parasutterella excrementihominis* differed significantly between SAD patients and controls. Additionally, functional differences were evident with the microbiome of SAD patients enriched for the gut metabolic pathway, aspartate degradation I.

Strikingly, we found *Anaeromassilibacillus sp An250* to be present in almost half of our SAD group but in only one healthy control. *Anaeromassilibacillus* is a newly-discovered genus, which was first isolated in 2017 from the faecal sample of a 1-yo Senegalese patient with kwashiorkor [61]. Several strains of *Anaeromassilibacillus*, including *sp An250* have since been identified from the caecal microbiome of chickens [62, 63]. *Anaeromassilibacillus* is a member of the Clostridiales order and Clostridiaceae family of bacteria [61], taxonomic groups which are increased in the gut microbiota of patients with autism spectrum disorder (ASD) [64], depression [65] and schizophrenia [66]. Conversely, various genera from the Clostridiales order were found to be reduced in the faecal microbiota of people with GAD [21] although Clostridiales was positively correlated with anxiety scores in a study analysing serum microbial DNA composition in patients with mood disorders [67]. Despite such inconsistencies, significant shifts in the abundance of Clostridiales taxa appears to be common to many psychiatric disorders and may represent disease-shared microbial responses [68]. Furthermore, preclinical studies suggest a link between Clostridiales and social behaviour. In a recent study, mice subjected to early life social isolation stress showed a significantly increased abundance of Clostridiales. These mice subsequently demonstrated reductions in sociability and social novelty behaviours, which negatively correlated with Clostridiales abundance [69]. In another study, mice exposed to a social stressor had increased relative abundance of the genus Clostridium [70]. However, it is difficult to extrapolate findings from animal studies to humans [71] especially with regards to a process as complex as social behaviour.

Given the very recent addition of *Anaeromassilibacillus* to human microbiome databases, there is little in the existing literature about its role in human health and disease. It was one of several genera found to be enriched in untreated patients with MDD compared to those receiving antidepressant treatment, suggesting that it could be altered by psychotropic medication or be an indicator of treatment response [72]. Additionally, the relative abundance of *Anaeromassilibacillus* reduced significantly in the faeces of children with ASD after guar gum prebiotic supplementation, which was associated with reduced irritability and improved constipation [73], again suggesting that reduction of this genus may be associated with improved psychopathology. Thus, although the literature is sparse, *Anaeromassilibacillus* appears be of relevance in ASD and depression, psychiatric conditions which are highly comorbid with SAD [74, 75] and which may involve shared pathophysiological processes. Of note, we did not see a difference in the relative abundance of *Anaeromassilibacillus* in medicated, compared to, unmedicated SAD patients; although given the small sample size, this should be interpreted with caution.

*Gordonibacter* is another genus about which relatively little is known. It is a member of the *Eggerthellaceae* family and Coriobacteriia class [76] and is notable in its ability to produce urolithins from the metabolism of polyphenols [77], which may have an impact on mental health [78]. *Parasutterella* has been more extensively studied. It is a member of the *Sutterellaceae* family and in humans, is largely represented by a single species, *Parasutterella excrementihominis* [79]. Similar to our finding of lower *Parasutterella* levels in SAD, members of this genus have also been found to be reduced in ASD [80]. Weight and dietary factors appear to be important influences. *Parasutterella* is negatively associated with BMI and waist circumference [81] and conversely, can be induced by high sugar [82] and high-fat diets [83]. Our SAD group had elevated sugar intake and did not differ in terms of fat intake but, although the group difference for *Parasutterella excrementihominis* remained after adjusting for BMI, increased BMI in the SAD group could contribute to its reduced abundance in SAD patients.

It is difficult to interpret the importance and relevance of specific bacterial taxa differences in a patient group. The gut microbiome is a highly complex and dynamic ecosystem where microbes continuously interact with, and impact, one another and the host [84]. Attempts are underway to characterise microbial community structures and gain insights into the many complex microbe-microbe and host-microbe networks and interactions [85, 86]. Some human gut microbial groups appear to be highly influential and exert a metabolic impact on a substantial number of other microbial entities, so-called ‘network influencers’ [85]. None of our differentially expressed genera or species have been reported as being such core taxa or ‘influencers’ and it is unclear what these over- and under-represented taxa mean in the overall context of the gut microbial environment of SAD patients. With this in mind, exploring microbial function may offer deeper insights than relying on composition alone in an ever-changing ecosystem.

Using GBMs and GMMs, which are microbiome-related functional pathways that have been manually curated from existing literature [32, 33], we identified one functional pathway that was enriched in the SAD group – aspartate degradation I. According to MetCyc, a comprehensive reference database of metabolic pathways and enzymes [87], this pathway involves the conversion of the amino acid, L-aspartate to the corresponding keto acid, oxaloacetate, by the enzyme, aspartate aminotransferase (AspAT). Several bacteria and archaea have demonstrated this enzymatic ability including *Haloferax mediterranei* [88], *Pseudoalteromonas translucida TAC125* [89], *Saccharolobus solfataricus* [90] and *Escherichia coli* [91, 92]. Interestingly, bacterial AspAT enzyme activity may represent a link between gut microbiome function and the tryptophan-kynurenine pathway, a key physiological system in psychiatric disorders. There are significant interactions between tryptophan metabolism and the MGB axis [93–96] and the gut microbiome may influence host diet selection behaviour by mediating the availability of essential amino acids such as tryptophan [97]. Tryptophan metabolism involves the downstream conversion of kynurenine to kynurenic acid (KYNA) by the enzyme kynurenine aminotransferase (KAT). KYNA is an important neuroactive substance, which is elevated by chronic stress in animal models [98, 99] as well as in psychiatric conditions such as schizophrenia [100, 101] and SAD [102]. Notably, KAT activity has been detected in *E. coli* cells in vitro, and authors suggested that the source of KYNA detected in the rat small intestine could be the gut bacteria [103]. This bacterial KAT enzyme protein has been identified as being identical to the bacterial AspAT enzyme [104] and thus, an elevation in the ‘aspartate degradation I’ functional pathway may represent increased KAT, as well as AspAT potential, by the microbiome. While currently a hypothetical supposition, it is possible that the elevated peripheral KYNA which we previously reported in SAD patients [102] may be linked with the key functional difference seen in the microbiome of this group. In support of this hypothesis is the fact that D-cycloserine, an orally-administered, broad-spectrum antibiotic, has been found to enhance the treatment response to exposure therapy for SAD [105, 106], an effect which could plausibly be related to its ability to inhibit KAT activity and lower KYNA [107].

This is, to our knowledge, the first study to investigate the composition and function of the gut microbiome in patients with SAD and has several notable strengths. Firstly, our sample consisted of carefully selected patients with a pre-existing clinical diagnosis of SAD who had sought treatment from a mental health professional. Secondly, we used a whole genome shotgun sequencing method, providing information on the functional capacity of the microbiome, as well as offering greater resolution of bacterial species identification, than the more commonly used 16 S rRNA gene sequencing [108, 109]. Thirdly, we took into account many of the important host variables known to confound gut microbiota studies in human disease [110]. Stool quality is a particularly strong source of gut microbiota variance [110, 111] which has often been neglected in psychiatric microbiota studies. Stool consistency, as measured by the BSC, was matched between our groups, as were other important variables, including age, sex, birth delivery mode, smoking status and alcohol. Our groups were not matched in terms of BMI and exercise levels, variables which may be of relevance to the gut microbiome [112, 113]. Although adjusting for these variables did not affect group differences, it would, of course, be preferable to have samples with equivalent BMI and exercise scores. Additionally, we collected detailed dietary information, which has often been lacking in studies of the microbiota in psychiatric conditions. Our groups were well-matched in terms of overall dietary intake. The only difference seen was in relation to carbohydrate consumption, driven by higher sugar intake in the SAD group, and this was adjusted for in our statistical analyses.

Study limitations include the small sample size and the single-time point nature of the study, which prevents the establishment of any causal relationships. Additionally, two thirds of our SAD patients were taking psychotropic medication, which may have had an impact on microbiota composition [114, 115]. The majority of our medicated patients were taking an SSRI antidepressant, escitalopram being the most commonly prescribed. Escitalopram has antibacterial activity against some gut commensal strains in vitro [116, 117] although this effect did not translate to an in vivo animal model [117]. Other prescribed SSRIs in our patient group included fluoxetine, citalopram, sertraline, paroxetine and vortioxetine, all of which have shown varying levels of antibacterial activity in vitro [117–120], with in vivo evidence available for fluoxetine [121–123]. The SNRI venlafaxine, conversely, does not appear to impact common gut commensals in vitro [116, 117], although an influence on the microbial richness and on the abundance of certain genera were seen in a mouse model [122]. Thus, the translatability of studies using isolated in-vitro strains to animal models is unclear, with even more uncertainty in relation to their applicability to the human gut microbiome. Limited human data in relation to the effect of antidepressants on the microbiome is available. A small study of 17 depressed patients commenced on escitalopram, found no significant differences in beta-diversity or in any taxa levels between pre-treatment and 6-week post-treatment time-points, although increased alpha diversity was evident [124]. Furthermore, a longitudinal study of 40 patients with depression and/or anxiety revealed no difference in beta diversity between those taking, and not taking, antidepressant medications and no change in alpha diversity in antidepressant-treated patients between baseline and endpoint timepoints. Antipsychotic medications, conversely, did appear to exert an effect on the gut microbiome [125], consistent with previous findings [126]. Two of our patients were prescribed low-dose Quetiapine, a second-generation antipsychotic which thus may have had an impact.

Aside from impacting microbiota composition, it is also of course possible that psychotropic medications could have influenced functional pathways. A recent study demonstrated that oral intake of fluoxetine or amitriptyline by rats exposed to chronic unpredictable mild stress resulted in alterations in KEGG metabolic pathways, particularly those pathways concerning carbohydrate metabolism, membrane transport, and signal transduction [127]. However, no such alterations in KEGG pathways were seen in a longitudinal follow-up of psychiatric patients taking antipsychotics, antidepressants and/or anxiolytic medications [125]. In an approach similar to ours, these authors also chose to analyse GBMs in the psychiatric group, although they specifically investigated only 6 of the 56 available GBMs, namely those involving GABA and tryptophan synthesis or degradation. They found alterations in certain GBMs in patients taking antipsychotics and antidepressants but not in those taking anxiolytic medications. All in all, although there is clear evidence that many antidepressants have antibacterial effects, this evidence is based primarily on in-vitro and animal studies, and the impact of these medications on the human gut microbiome structure and function remain largely unknown. Although we did not find any differences in the relative abundance of any taxa between medicated and unmedicated patients, we cannot rule out a potential influence.

Finally, some of our SAD patient group had a past history of depression and/or a comorbid anxiety disorder. However, patients with a current depressive episode were excluded and in all, the primary diagnosis was SAD with any comorbid anxiety disorder representing a secondary diagnosis. Although we did not find a difference in the relative abundance of any taxa in those SAD patients with or without a past history of MDD, this must be interpreted with caution given the small numbers of such sub-groups. While it is not possible to disentangle the currently reported observations from the past psychiatric history of study participants, this was a clinically representative sample and we believe that including such patients makes our findings more generalizable considering the significant overlap between depression and anxiety disorders. Given the paucity of studies exploring the gut microbiome in any clinical anxiety disorders, our findings, despite the limitations, are important in generating a foundational base for larger, prospective and interventional microbiome studies in these highly prevalent and disabling psychiatric conditions. Additionally, future preclinical studies and secondary validation experiments would offer a complementary approach to confirm the presence and role of these differentially expressed bacterial taxa and functional pathways. Earlier compositional microbiota studies in depression [60, 128] and ASD [129, 130] have paved the way for a variety of successful interventional studies utilising probiotics [25], dietary change [131] and faecal microbiota transplantation (FMT) [132] in these conditions. It is hopeful that microbiota-based therapeutic interventions may also be realised for patients with clinical anxiety disorders. Indeed, a previous cross-sectional study in university students has suggested that consumption of fermented foods may be protective against the development of social anxiety [31].

In conclusion, the gut microbiome of patients with SAD differs in composition and function to that of healthy controls, raising the possibility that the MGB axis may represent a biomarker reservoir and potential therapeutic target for this early-onset, chronic disorder. Further preclinical studies focussing on mechanistic pathways and larger, longitudinal studies in SAD patients are needed to validate our preliminary results, understand the clinical implications (if any) and investigate the impact of psychotropic medication and treatment on the gut microbiome in SAD.

## Data Availability

Whole genome sequences are available at: European Nucleotide Archive, Accession ID PRJEB58864.
